# SERS-Based Microneedle Biosensor for In Situ and Sensitive Detection of Tyrosinase

**DOI:** 10.3390/bios14040202

**Published:** 2024-04-19

**Authors:** Zimeng Gu, Di Zhao, Hongyan He, Zhenhui Wang

**Affiliations:** 1 Frontiers Science Center for Materiobiology and Dynamic Chemistry, Engineering Research Center for Biomedical Materials of Ministry of Education, East China University of Science and Technology, Shanghai 200237, China; guzimeng333@163.com (Z.G.); zhaodi2078@163.com (D.Z.); wangzhenhui9999@163.com (Z.W.); 2 Engineering Research Center for Biomedical Materials of Ministry of Education, East China University of Science and Technology, Shanghai 200237, China; 3 School of Materials Science and Engineering, East China University of Science and Technology, Shanghai 200237, China

**Keywords:** SERS, tyrosinase, microneedle, in situ detection

## Abstract

Tyrosinase (TYR) emerges as a key enzyme that exerts a regulatory influence on the synthesis of melanin, thereby assuming the role of a critical biomarker for the detection of melanoma. Detecting the authentic concentration of TYR in the skin remains a primary challenge. Distinguished from ex vivo detection methods, this study introduces a novel sensor platform that integrates a microneedle (MN) biosensor with surface-enhanced Raman spectroscopy (SERS) technology for the in situ detection of TYR in human skin. The platform utilized dopamine (DA)-functionalized gold nanoparticles (Au NPs) as the capturing substrate and 4-mercaptophenylboronic acid (4-MPBA)-modified silver nanoparticles (Ag NPs) acting as the SERS probe. Here, the Au NPs were functionalized with mercaptosuccinic acid (MSA) for DA capture. In the presence of TYR, DA immobilized on the MN is preferentially oxidized to dopamine quinone (DQ), a process that results in a decreased density of SERS probes on the platform. TYR concentration was detected through variations in the signal intensity emitted by the phenylboronic acid. The detection system was able to evaluate TYR concentrations within a linear range of 0.05 U/mL to 200 U/mL and showed robust anti-interference capabilities. The proposed platform, integrating MN-based in situ sensing, SERS technology, and TYR responsiveness, holds significant importance for diagnosing cutaneous melanoma.

## 1. Introduction

Melanoma is a malignant tumor arising from melanocytes and is one of the most aggressive forms of skin cancer [1]. In the early stages of melanoma development, surgical excision can achieve a high cure rate [2]. Current diagnosis of melanoma primarily relies on clinical assessment by physicians and histopathological examination [3]. Therefore, the identification of a biomarker for the swift and sensitive detection of early melanoma is deemed essential.

Tyrosinase (TYR), a copper-containing polyphenol oxidase that regulates melanin synthesis, has been identified as a candidate biomarker for melanoma due to its overexpression in melanoma cells and accumulation in skin cells, which correlates with the severity of malignancy [4]. To date, diverse methods for TYR detection have been developed, including colorimetric [5,6], liquid chromatography [7], electrochemical [8,9], and fluorescence techniques [10]. Nonetheless, these techniques persist with limitations such as low sensitivity, complex procedures, high equipment costs, susceptibility to non-specific substance interference, and fluorescence background interference. Consequently, the development of highly sensitive, facile, selective, and stable methods for TYR detection remains a significant challenge.

Surface-enhanced Raman spectroscopy (SERS) has demonstrated substantial potential in the fields of biomedicine, chemical analysis, and materials science due to its remarkably high sensitivity, selectivity, non-destructiveness, and resistance to photobleaching [11,12,13]. In biomedical applications, SERS mainly uses two methods: labeling and label-free techniques [14,15,16,17]. The label-free technique provides biomolecule-specific vibrational data through sample–substrate interaction, offering intrinsic fingerprint information, though it can be affected by impurities [14,18,19]. The labeling method uses intense Raman reporter molecules as tags, enhancing accuracy and allowing for semi-quantitative analysis, but with more complex procedures [20,21].

To date, numerous studies have employed SERS technology for the detection of TYR. Lu et al. [22] reported a method for quantitative TYR detection using magnetic beads, with 4-mercaptobenzonitrile (4-MB)-modified gold nanoshells serving as Raman reporters and functionalized beads for substrate capture. TYR activity was detected through alterations in residual SERS tag concentrations. Challenges included non-uniform antibody modification on magnetic surfaces, a complex and lengthy labeling process, and the need for expensive reagents. Wang et al. [23] developed a novel, portable, and recyclable SERS sensor for TYR activity, utilizing gold nanoparticles and para-thiolcatechol (p-TC) on ITO electrodes to monitor TYR-catalyzed p-TC conversion via SERS. Despite the complex p-TC synthesis and potential signal interference in complex media, this approach offered a means to assess TYR activity, albeit with implications for detection sensitivity and specificity. However, the aforementioned tests were conducted ex vivo on human serum samples, which can cause patient discomfort and tissue damage during the sampling process; more significantly, TYR levels in blood are typically low in the early stages of disease. In the study of Zhang et al. [24], no significant statistical differences in TYR levels were observed between serum samples from melanoma patients and healthy volunteers. Given that cutaneous melanoma predominantly occurs on the skin surface, highly sensitive detection of TYR concentrations in the skin is of critical importance for early clinical screening of melanoma. However, achieving minimally invasive and effective sensitive detection of the true TYR levels in target skin areas remains a significant challenge.

Microneedles (MN), as an emerging biosensing technology, employ minuscule needle-like structures for minimally invasive transdermal detection, offering near real-time molecular-level specificity [25,26,27,28]. Recognized for its minimal tissue impact, swift recovery time, and patient-friendly nature, microneedle technology has attracted significant interest and has emerged as a promising avenue for accurate clinical diagnostics and health monitoring. It also demonstrates considerable potential in the detection of subcutaneous biomarkers, efficiently tracking a range of skin-underlying biomarkers such as glucose, lactate, neurotransmitters, cholesterol, glycine, and pH through a user-friendly, swift, and non-invasive approach [29,30,31,32,33,34].

**Figure 1:**
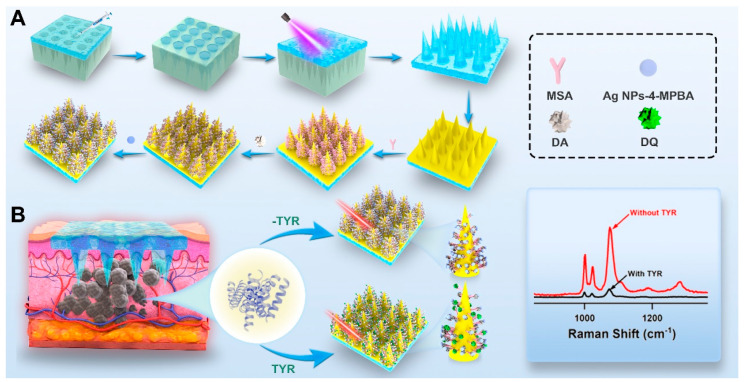
(**A**) Schematic illustration of the construction procedure for the MN. (**B**) The principle of SERS microneedle biosensor for in situ detection of TYR.

In this study, we developed a microneedle-based SERS sensor for in situ detection of TYR to facilitate rapid and effective clinical screening for melanoma. As depicted in Figure 1A, gold nanoparticles (Au NPs) were assembled on the microneedle array, and the Au NPs were modified with thioglycolic acid (MSA) to capture dopamine (DA) through amide reactions between functional carboxylate (–COOH) and amine (–NH_2_) groups. Silver nanoparticles (Ag NPs) modified with 4-mercaptophenylboric acid (4-MPBA) served as the SERS probe, forming stable cyclic boronates through interactions between the boronic acid group of 4-MPBA and hydroxyl groups. When the microneedles penetrate skin containing TYR, DA is oxidized to dopamine quinone (DQ), disrupting the interaction between Ag NPs-4-MPBA and the microneedle array, leading to a decrease in SERS signal, as shown in Figure 1B. The intensity of the SERS signal is inversely proportional to the increase in TYR concentration, allowing for the detection of TYR levels.

## 2. Materials and Methods

### 2.1. Materials and Reagents

Hydrogen tetrachloroaurate (HAuCl_4_·3H_2_O), polydimethylsiloxane (PDMS), ascorbic acid (AA), and methacrylate (PMMA) were purchased from Sigma-Aldrich. Hexadecyl trimethyl ammonium bromide (CTAB), sodium borohydride (NaBH4), hexadecyl trimethyl ammonium chloride (CTAC), sodium hydroxide (NaOH), polyvinyl pyrrolidone (PVP), dichloromethane (CH_2_Cl_2_), mercaptosuccinic acid (MSA), methylthiazolyldiphenyl-tetrazolium bromide (MTT), silver nitrate (AgNO_3_), agarose, hexyl hydride, and dimethyl sulfoxide (DMSO) were purchased from Aladdin (Shanghai, China) Co., Ltd. 4-mercaptophenylboronic acid (4-MPBA) was purchased from MacLean Biochemical Co., Ltd. (Shanghai, China). DMEM and FBS were purchased from Gibco (New York, NY, USA). Tyrosinase (TYR) was purchased from MedChemExpress (Shanghai, China). Antibiotics were purchased from Thermo Fisher Scientific (Shanghai, China). Male C57 mice at 6 weeks old were purchased from Shanghai Shengchang Bio-Tech Pty., Ltd. (Shanghai, China). 

### 2.2. Synthesis of Au NPs

Au NPs were produced utilizing a method reported by Zheng et al. [35], which could precisely control the diameters by varying the sizes of the seeds. In brief, a 10 mL aqueous mixture containing HAuCl_4_ (0.25 mM) and CTAB (0.1 M) was thoroughly mixed. Then, a NaBH_4_ solution (10 mM, 0.6 mL) was introduced into the mixture. After 2 min of vigorous stirring, the mixture was allowed to settle at a temperature of 27 °C for a duration of 2 h to form the initial seed crystals. For further synthesis of the growth solution, the initial seeds, along with aqueous solutions of CTAC (0.2 M, 4 mL) and AA (0.1 M, 3 mL), were mixed in a vial. Afterward, a HAuCl_4_ solution (1 mM, 6.5 mL) was introduced into the mixture and incubated for 20 min at 27 °C. The product was collected by centrifugation at 12,000 rpm for 30 min and dispersed in CTAC aqueous solution (20 mM, 2 mL) following thorough washing. This dispersion was employed as 10 nm seed crystals for the subsequent growth cycle.

A combination of CTAC (0.1 M, 2 mL), AA (10 mM, 130 μL), and the 10 nm seeds (50 μL) was prepared. Subsequently, an aqueous solution of HAuCl_4_ (0.5 mM, 2 mL) was slowly introduced into the mixture at a rate of 2 mL/h. Afterward, the mixture underwent centrifugation at 12,000 rpm for 30 min and was then redispersed with ultrapure water. The final product, the 30 nm Au NPs, was then stored at 4 °C for subsequent applications. 

### 2.3. Synthesis of Ag NPs and Ag NPs-4MPBA

Ag NPs were synthesized based on the Lee and Meisel method [36]. Specifically, AgNO_3_ (18 mg) was dissolved in DI water (100 mL), and sodium citrate solution (1%, 5 mL) was introduced. The mixture was heated to boiling while stirring for 1 h, then cooled and refrigerated at 4 °C for subsequent use. 4-MPBA (8 mM, dissolved in 0.2 M NaOH, 5 mL) was added to the Ag NPs solution (1 mL), and the mixture was stirred at ambient temperature for a duration of 1 h to facilitate the reaction. The mixture was then centrifuged and resuspended for further applications.

### 2.4. Synthesis of the Au-Coated PMMA MN Array

The PMMA MN array was prepared via a simple micromolding method. In brief, the microneedle model was designed using 3DsMax software (2020), followed by the fabrication of a master mold with a micro-milling device. This master mold was then employed to produce a reusable PDMS MN mold. An array of PMMA was fabricated by casting the PMMA solution into a designed PDMS mold; then, the mold with PMMA on top was put into a vacuum dryer to remove air bubbles. The prepolymer was then solidified via UV irradiation at a wavelength of 365 nm for a period of 1.5 min. The resulting MN array was carefully separated from the PDMS mold. The array underwent cleaning with ethanol and DI water, followed by drying in a vacuum oven set at 40 °C. Subsequent processing steps are delineated in Figure 1A. Then, the Au-coated MN arrays were produced by Marangoni effect-driven transfer and compression at three-phase interfaces according to Lin et al. [37]. Briefly, the MN patch was sonicated in anhydrous ethanol for 20 min and dried at 40 °C to eliminate organic residues and oil contaminants from its surface.

Then, Au NPs dispersion (8 mL) was subjected to centrifugation twice to pellet the nanoparticles, followed by the removal of the supernatant. And the sediment at the bottom was re-suspended in a PVP solution (1 wt%, dissolved in ethanol, 2 mL) with vigorous stirring. The resulting solution was then transferred to a 1.5 mL centrifuge tube. After a second round of centrifugation, the sediment was re-suspended in ethanol (1 mL) and stored at 4 °C for future applications. For the assembly of CTAC-coated nanoparticles, a solution of PVP-stabilized nanoparticles (100 μL) was mixed with dichloromethane (1 mL) in a 5 mL centrifuge tube. Following this, water (2 mL) was introduced into the mixture, and the contents were vigorously mixed for 30 s to promote thorough interaction between the nanoparticles in the organic phase and the aqueous phase. In less than 2 min, a gold film formed at the interface of the water and CH_2_Cl_2_. Subsequently, hexane (400 μL) was slowly added along the container wall, facilitating the migration of nanoparticles towards the interface between the aqueous phase and hexane, resulting in the formation of a densely packed nanoparticle monolayer. Once the surplus hexane had been eliminated, the MN array was immersed into the layer of gold film that had precipitated along the tube’s wall. The gold film was gently peeled off from the underlying surface and transferred onto a substrate, thereby fabricating the arrays of Au NP-MN.

### 2.5. Synthesis of Functionalized MN Arrays for SERS

First, the surfaces of the Au NP-MN arrays were functionalized with MSA through the formation of Au-S bonds. For each set of prepared MN arrays, a solution of MSA (4 mL, 20 mM) was introduced into individual 12-well plates containing the cleaned Au NP-MN arrays and incubated for 4 h on an orbital shaker to introduce carboxyl (−COOH) functionalities onto its surface. After that, the MN arrays were subjected to rigorous washing with DI water to eliminate any nonspecifically bound MSA molecules. The carboxyl-modified MN array was then sequentially treated with EDC (10 mM) and NHS (10 mM) to activate the carboxyl groups. Next, the MSA-modified Au NP-MN arrays were immersed in freshly prepared DA solution (3 mg/mL) for 2 h, followed by washing with DI water and drying with nitrogen. Dopamine was covalently anchored to the MN array surface via a bond formed between the carboxyl groups of mercaptosuccinic acid and the amine groups (–NH_2_) of dopamine. Then, Ag NPs-4MPBA dispersion was added and incubated at 37 °C to obtain the microneedle SERS sensor, followed by washing with DI water for further use. The complete fabrication process of the microneedle patch is depicted in Figure 1A.

### 2.6. Preparation of Skin Phantoms

The skin phantoms were prepared according to the method developed by Yuen and Liu [38]. Briefly, agarose solution (3 wt%, 50 mL) was boiled. Then, the target analytes, i.e., R6G or TYR, were added into the agarose gel once it had cooled to 40 °C. This process was utilized to fabricate a series of calibration phantoms, each containing the test molecules at varying concentrations.

### 2.7. Cytotoxicity Assessment

The cytotoxicity of the MN platform was assessed with an MTT viability assay. Cells were plated at a concentration of 10^4^ per well in 96-well plates, each filled with 200 μL of DMEM (complete with 10% FBS and 1% antibiotics), and incubated at 37 °C in a 5% CO_2_ environment for 24 h. The MN platform was sterilized via 40 min of UV exposure and subsequently immersed in 200 μL DMEM. This suspension was then transferred to the 96-well plates for co-incubation with the cells, with five replicates per group. A control group was established using cells incubated solely with the medium. At 12 h and 24 h intervals, the medium was aspirated, and 20 μL of MTT solution was added to each well for 3 h of incubation. Subsequently, the medium was replaced with 200 μL DMSO, and the cells were incubated for an additional 15 min. The optical density (OD) was measured at a wavelength of 570 nm using a microplate spectrophotometer.

### 2.8. Characterizations

The size distribution and morphological characteristics of Au NPs were measured using transmission electron microscopy (TEM, JEM-2100F, JEOL Ltd., Tokyo, Japan). The morphology of MN was observed via scanning electron microscopy (SEM, Helios G4 UC, Thermo Fisher Scientific, Waltham, MA, USA). The mechanical characteristics of MN were evaluated using a dynamic biomaterials mechanical testing system (Electroforce 3230, TA Instruments, New Castle, DE, USA). The ultraviolet–visible absorption spectra were acquired with a UV–vis–NIR spectrophotometer (Lambda 950, Lambda, San Jose, CA, USA). The element composition was characterized by X-ray photoelectron spectroscopy (XPS, Thermo Scientific K-Alpha+, USA). The H&E staining and the immunohistochemical analysis of tissue was observed on an inverted microscope platform (Leica DMi8, Leica, Beijing, China). The cytotoxicity of the MN was assessed using a multifunctional microplate reader (SpectraMax M2, Molecular Devices, Shanghai, China).

### 2.9. Raman Measurements

The efficacy of SERS using the microneedle (MN) platform was evaluated in skin phantoms and animal skin tissue through a Raman microspectroscopy system (DXR3xi Raman Imaging Microscope, Thermo Fisher Scientific, USA). The detection mechanism of the SERS microneedle is illustrated in Figure 1B. The MN platform was utilized to penetrate skin phantoms and mouse skin, enabling laser illumination and Raman detection. A microscope objective (10×, NA = 0.25, Shibuya Optical Co., Ltd., Wako-shi, Japan) was used to concentrate 785 nm laser light onto a tip of the MN platform, with an emitted laser power of approximately 16.2 mW. The system was calibrated for optimal SERS signal enhancement. The spectra presented in subsequent figures were derived from raw data after baseline correction and fluorescence noise reduction.

## 3. Results and Discussion

### 3.1. Characterization of the MN

**Figure 2:**
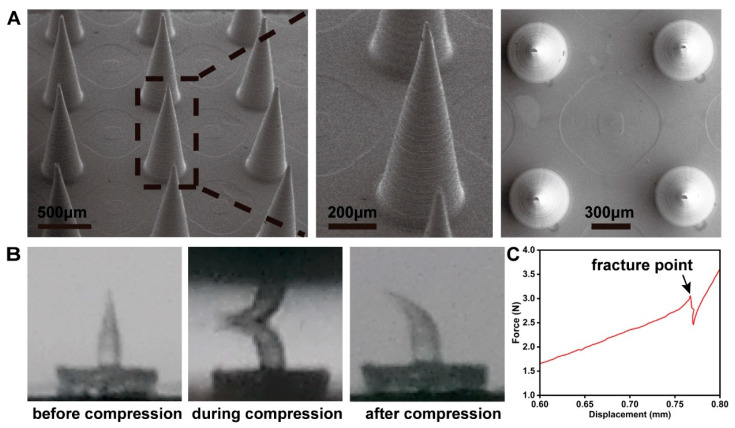
(**A**) SEM image of the fabricated MN array. (**B**) Optical image of the single-needle compression process. (**C**) Force–displacement curves of MN.

The constructed MN demonstrated a regular conical shape with an acute tip, measuring 1000 μm in height, 390 μm at the base diameter, and featuring a 1000 μm gap between the centers of adjacent MNs, as shown by SEM (Figure 2A). The mechanical characteristics of the MN were assessed through force–displacement curves. During compression, no severe brittle fractures were observed (Figure 2B), confirming that the MN was tough enough. This is advantageous for the insertion of the MN into skin, as it helps to prevent needle tip breakage and residue within the skin. The tip of a single needle fractured at a compression force of 3.05 N (Figure 2C), meeting the condition reported by Shawn et al. [39] that successful skin insertion occurs when the fracture force exceeds 0.1 N. 

**Figure 3:**
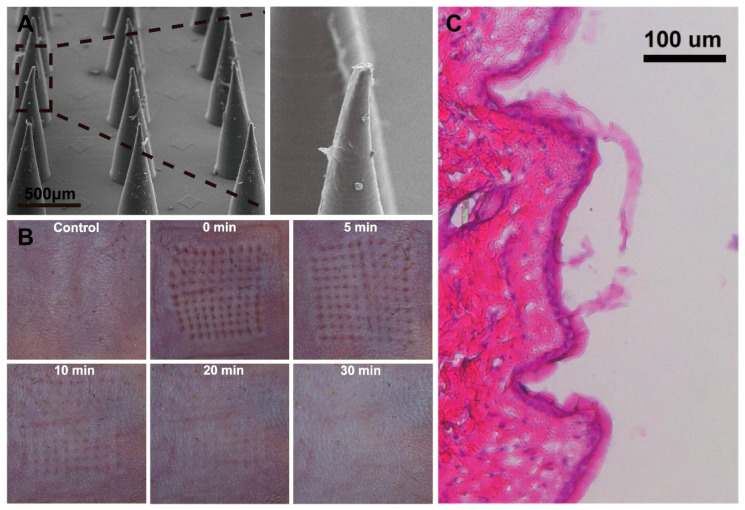
(**A**) SEM images of the MN after piercing mouse skin. (**B**) The mouse skin recovery dynamics after puncturing by the MN array. (**C**) The cross-section image of H&E-stained mouse skin.

The mechanical strength of the MN was evaluated by punching in mouse skin. Despite some tips bending slightly after penetration, the MN array remained intact throughout the testing process (Figure 3A). Thus, based on the aforementioned data, it was demonstrated that the MN with sufficient mechanical strength would penetrate the skin without fracturing. To investigate the recovery of the skin and the skin insertion depth, an in vitro insertion test was conducted on the abdominal areas of the mice. The MN patch (10 × 10) was pressed onto the mouse abdomen with a thumb and withdrawn after 5 min. After the removal of the MN patch, a pattern of microholes was observed on the mouse skin. These microholes, formed by the MN, nearly released within 20 min, and the skin area returned to its initial state within 30 min (Figure 3B), demonstrating low invasiveness to the skin. The swift restoration of the skin mitigates the risk of infection and pathogen ingress. Histological analysis, depicted in Figure 2C, reveals an approximate penetration depth of 100 μm, indicating that the MN effectively traverses the stratum corneum and epidermis while minimizing interaction with dermal blood capillaries and nerve endings, thereby facilitating a painless application process [40]. Consequently, based on the data presented, it is anticipated that an MN possessing sufficient mechanical strength will successfully penetrate the skin intact, without fracturing.

### 3.2. Optimization of the SERS Sensor

**Figure 4:**
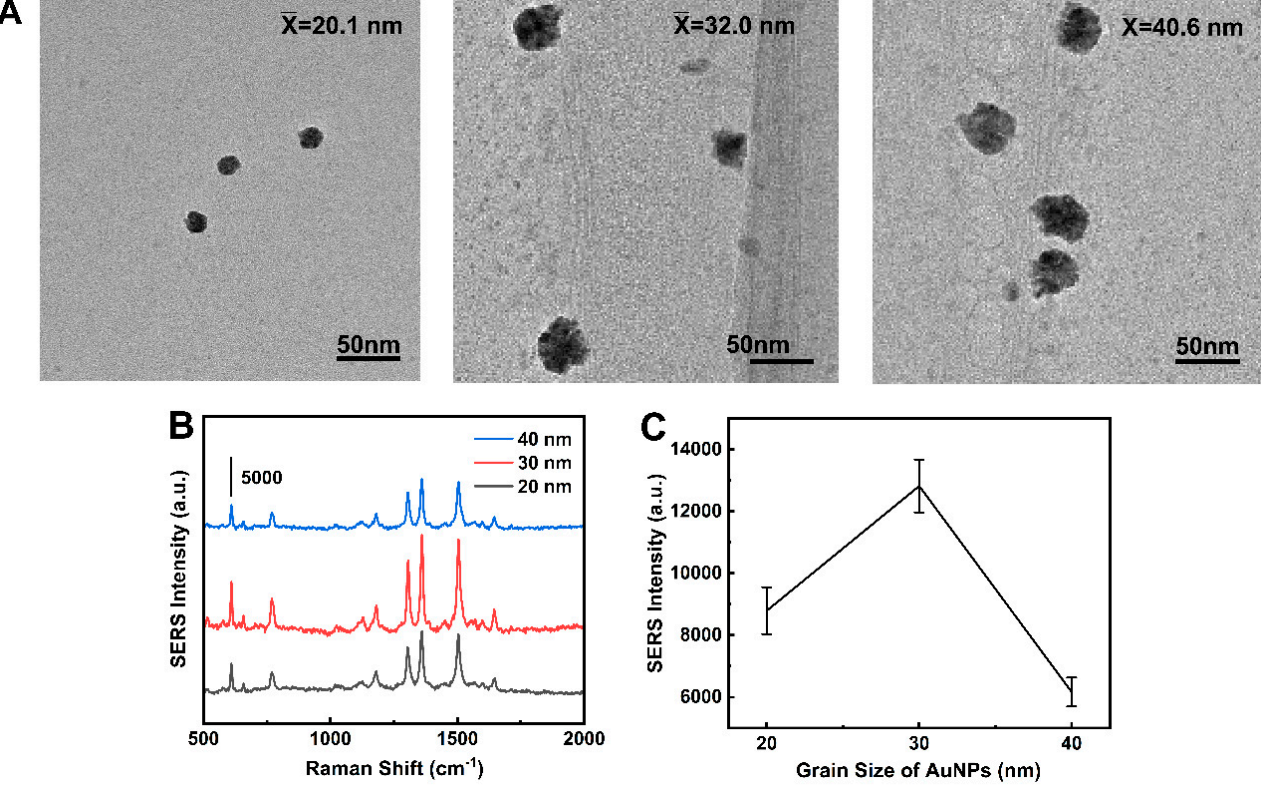
(**A**) TEM images of Au NPs (20, 30, 40 nm). (**B**) SERS spectra of MN modified with Au NPs (20, 30, 40 nm) using 10^−5^ mol/L R6G as the signal molecule. (**C**) Peak intensities of *I*_1360_ according to the spectra shown in panel (**B**).

**Figure 5:**
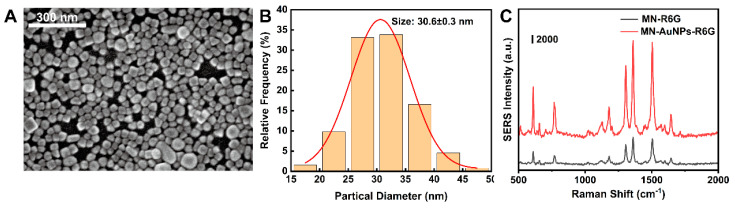
(**A**) SEM image of 30 nm Au NPs. (**B**) Au NPs particle size statistics. (**C**) SERS spectra of MN with 10^−5^ mol/L R6G as the signal molecule.

To optimize the performance of the MN sensor, we investigated these conditions, including the Au NPs size and the concentrations of MSA and 4-MPBA in the synthesis. Figure 4A demonstrates that Au NPs of 20 nm, 30 nm, and 40 nm diameters exhibit a consistent spherical morphology, in accordance with the anticipated experimental dimensions. Figure 4B,C illustrates that Au NPs with a diameter of 30 nm exhibit superior Raman enhancement effects. SEM image shows that Au NPs are densely packed, without obvious aggregates and large void spaces (Figure 5A). The particle size distribution is relatively uniform, conforming to a normal distribution with an average diameter of 30.6 ± 0.3 nm (Figure 5B). R6G was chosen as the Raman signal molecule to validate the SERS enhancement effect of Au NP-modified MNs [25]. Figure 5C confirms that the Au NP-modified MNs exhibited significant Raman enhancement, ascribed to the strong electromagnetic enhancement conferred by the uniformly and densely arrayed Au NPs.

**Figure 6:**
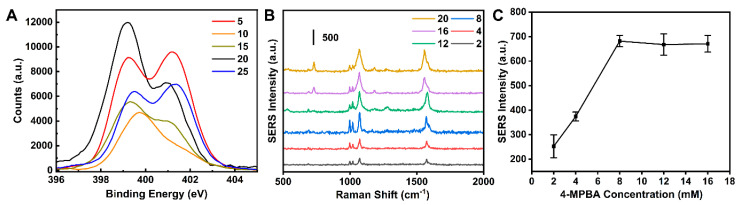
(**A**)XPS survey scan of MSA-modified MN at different concentrations (5, 10, 15, 20, and 25 mM). (**B**) Dependence of the SERS intensity of the MPBA-modified Ag NPs on the MPBA concentration used for the modification. (**C**) Peak intensities at 1072 cm^−1^ according to the spectra shown in panel (**B**).

The N content on the MN surface was highest at an MSA concentration of 20 mM, as shown by X-ray photoelectron spectroscopy (XPS) (Figure 6A). Subsequently, the Raman signal intensity at various 4-MPBA concentrations revealed no significant change once the 4-MPBA concentration reached 8 mM (Figure 6B,C). In summary, Au NPs exhibited a significant enhancement effect on Raman signals. Optimal Raman enhancement for the microneedle Raman sensor was achieved when the gold nanoparticles were at a diameter of 30 nm, with MSA and 4-MPBA concentrations set at 20 mM and 8 mM, respectively.

### 3.3. Feasibility of the MN SERS Sensor for TYR Detection

**Figure 7:**
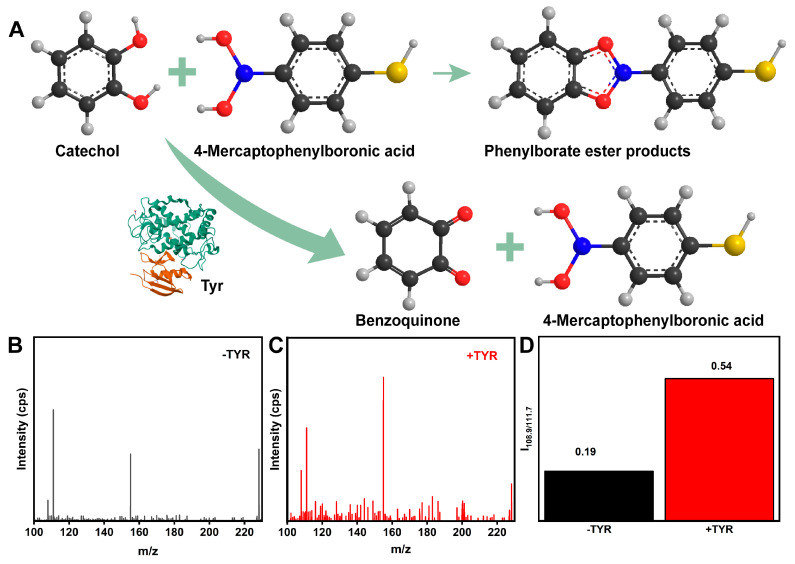
(**A**) Illustration of the operational mechanism for the TYR reaction. (**B**) HRMS image of the reaction product between 4-MPBA and catechol in the absence of TYR. (**C**) HRMS image of the reaction product between 4-MPBA and catechol in the presence of TYR (100 U/mL). (**D**) The intensity ratio between ionization fragment peaks at *m*/*z* = 108.9 and *m*/*z* = 111.7 after incubation with TYR.

The MN sensor was tested using high-resolution mass spectrometry (HRMS) to assess the interaction between 4-MPBA and catechol to validate the feasibility of the reaction between 4-MPBA and dopamine (Figure 7A). The representative electrospray ionization fragment at *m*/*z* = 228 for the new product indicated that the reaction between boric acid and catechol occurred successfully. After incubation with TYR, the intensity ratio of the ionization fragment peaks at *m*/*z* = 108.9 (benzoquinone) and *m*/*z* = 111.7 (catechol) increased by approximately twofold (Figure 7B–D), signifying the catalytic conversion of catechol to benzoquinone by TYR and a substantial increase in the benzoquinone content. Notably, the pronounced rise in the fragment ion peak at *m*/*z* = 153.99, corresponding to 4-MPBA, corroborated the conversion of catechol to benzoquinone, which is non-reactive with 4-MPBA, resulting in 4-MPBA accumulation. Furthermore, the sharp reduction in the intense fragment ion peak at *m*/*z* = 228 confirmed that the benzoquinone generated by TYR inhibited the formation of borate ester products. The aforementioned experimental results demonstrate that, during the oxidation of catechol, TYR also disrupts the five-membered ring boronate ester structure formed between catechol and 4-MPBA, further validating the feasibility of the biosensor.

### 3.4. Development for the Standard Working Curve of TYR

**Figure 8:**
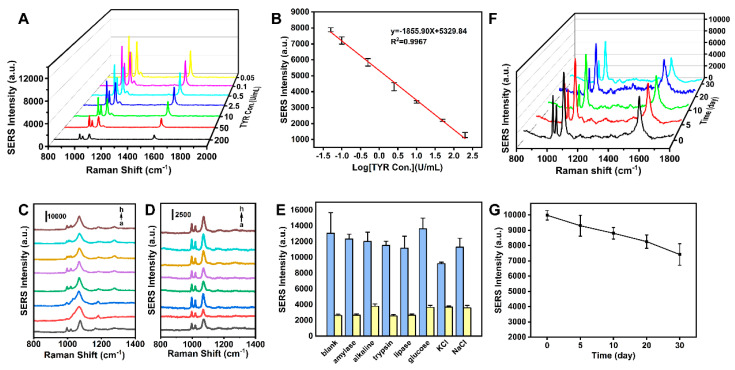
(**A**) SERS spectra of MN after reaction with various concentrations of TYR. (**B**) Line relationship between the Raman intensity at 1072 cm^–1^ and the concentration of TYR. (**C**) SERS spectra of the sensor in the absence and presence of different biologically relevant species: (a–h) blank, amylase, alkaline, trypsin, lipase, glucose, KCl, and NaCl. (**D**) SERS spectra of the sensor in the presence of TYR and diverse relatively biological interferents. (**E**) Peak intensities at 1072 cm^−1^ with the blue histogram and yellow histogram were obtained according to the spectra shown in panels (**C**,**D**). (**F**) SERS spectra of MN for different days of placement (0, 5, 10, 20, and 30 days). (**G**) Peak intensities at 1072 cm^−1^ according to the spectra shown in panel (**F**).

The MN sensor’s potential for TYR detection was further validated through the assessment of its application at various TYR concentrations under previously optimized conditions, leading to the formulation of a standard working curve. Oxidation of dopamine (DA) to dopamine quinone (DQ) occurred in the presence of TYR, resulting in a reduced content of Ag NPs-4MPBA on the MN. Consequently, an inverse correlation was observed between the concentration of TYR and the SERS intensity of 4-MPBA. Subsequently, the MN was inserted into the skin phantoms containing varying concentrations of TYR for in situ SERS sensing, the SERS signal decreased as the concentration of TYR increased (Figure 8A). Figure 8B illustrates a strong linear correlation between the SERS intensity at 1070 cm^−1^ and TYR concentrations spanning 0.05 to 200 U/mL (R^2^ = 0.9967). Therefore, the MN-based sensing technique possesses considerable promise for the future detection of TYR.

### 3.5. Interference and Selectivity Studies

Furthermore, the selectivity of the MN platform for TYR detection was assessed by monitoring its responses to the target TYR and various biological interferents, including amylase, alkaline, trypsin, lipase, glucose, KCl, and NaCl, under identical conditions. As shown in Figure 8C and the blue histogram in Figure 8D, a distinct decrease in the SERS signal was observed exclusively with the target TYR, while the SERS signal showed no obvious changes for non-target molecules. Critically, a marked decrease was observed for TYR at 1070 cm^−1^, regardless of the presence of interfering substances (Figure 8D, yellow histogram in Figure 8E), primarily due to the specificity of the TYR-catalyzed strategy, where catechol oxidation by other coexisting skin components is of low probability. Thus, the proposed SERS sensor exhibited excellent resistance to interference, offering significant potential for the determination of TYR activity in authentic samples.

### 3.6. Stability Assessment

To assess the temporal stability of the MN sensor, the same batch of samples was stored at 4 °C for 0, 5, 10, 20, and 30 days, with Raman signals of 4-MPBA measured at each interval. A gradual decrease in the Raman peak intensity at 1070 cm^−1^ was observed over time, with the MN sensor’s response dropping to 7400 a.u. after 30 days (Figure 8F,G), which is adequate for subsequent detection in animal disease models.

### 3.7. Comparison of SERS Results with ELISA Analysis

**Figure 9:**
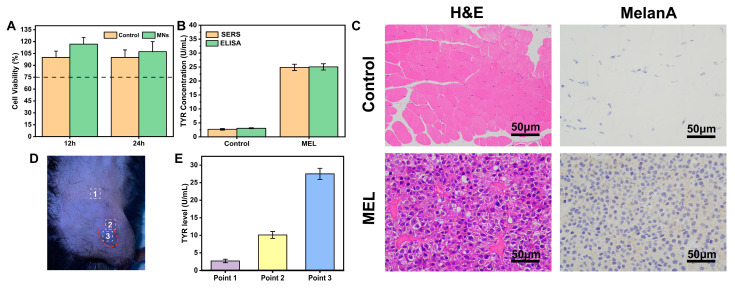
(**A**) Viability of C2C12 cells incubated with MN for different durations. (**B**) TYR levels of control and melanoma mice detected by SERS and ELISA. (**C**) H&E staining and immunohistochemistry of control and melanoma mice. (**D**) Photograph of MN sticking on different points of the mouse skin surface. (**E**) TYR levels on different points of the mouse skin surface. Point 1 sampled in normal skin; Point 2 sampled at edge of melanoma; Point 3 sampled in the center of melanoma.

To assess the MN platform’s utility, ELISA was employed to quantify TYR levels in both normal and melanoma-afflicted mice (Figure 9B). The results exhibited a TYR concentration of 3.1 ± 0.2 U/mL in normal mice and 25.1 ± 1.1 U/mL in melanoma mice, values which are generally consistent with the SERS results. Additionally, the MN exhibited no significant in vitro toxicity (Figure 9A). Consequently, its non-toxic, painless, and user-friendly characteristics render MN an appropriate candidate for the further investigation of transdermal TYR assessments.

### 3.8. In Situ Skin Model for TYR Detection

The immunohistochemical analysis of paraffin-embedded mice melanoma tissue labeling with MelanA further revealed the high expression of TYR levels in melanoma (Figure 9C), concurrently affirming the successful development of the MEL mouse model. Additionally, SERS signals from MNs placed within the melanoma region (melanoma region) were contrasted with those from the surrounding normal skin. Figure 9D,E demonstrate that TYR levels were more pronounced in the melanoma areas compared to their periphery and were virtually undetectable in normal skin, indicating the platform’s potential for distinguishing melanoma from healthy skin.

## 4. Conclusions

In essence, a novel, portable SERS sensor, utilizing Au NPs, was developed for the detection of a melanoma-related biomarker (TYR). This study offers a novel approach for in situ TYR analysis in skin, serving as an appealing alternative to prior serum-based assays for melanoma screening and diagnosis. The SERS sensor utilizes DA-functionalized Au NPs as the capture microneedle and 4-MPBA-modified Ag NPs as the SERS probe. Upon TYR exposure, DA is oxidized to DQ, leading to a reduced concentration of SERS probes on the sensor. TYR activity is monitored through the alteration in the intensity of the phenylboronic acid signal. This proposed MN platform demonstrated a robust linear correlation for TYR detection, achieving a low detection limit of 0.05 U/mL, attributed to the integration of MN-based in situ screening and targeted TYR sensitivity. In contrast to alternative approaches, the proposed method eschews intricate nanofabrication and costly biological recognition components, offers ease of storage and portability, and features a straightforward sensor platform preparation process. By tracking the temporal variation in TYR levels, this study provides new opportunities for the monitoring and diagnosis of melanoma.

## Data Availability

Data are contained within the article.
